# Binding of Citreoviridin to Human Serum Albumin: Multispectroscopic and Molecular Docking

**DOI:** 10.1155/2015/162391

**Published:** 2015-04-21

**Authors:** Haifeng Hou, Xiaolan Qu, Yuqin Li, Yueyue Kong, Baoxiu Jia, Xiaojun Yao, Baofa Jiang

**Affiliations:** ^1^School of Public Health, Shandong University, Jinan 250012, China; ^2^School of Public Health, Taishan Medical University, Taian 271016, China; ^3^School of Pharmacy, Taishan Medical University, Taian 271016, China; ^4^School of Chemistry and Chemical Engineering, Lanzhou University, Lanzhou 271000, China

## Abstract

Citreoviridin (CIT), a mycotoxin produced by *Penicillium citreonigrum,* is a common contaminant of wide range of agriproducts and detrimental to human and animal health. In this study, the interaction of CIT with human serum albumin (HSA) is researched by steady-state fluorescence, ultraviolet-visible (UV-Vis) absorption, circular dichroism (CD) methods, and molecular modeling. The association constants, binding site numbers, and corresponding thermodynamic parameters are used to investigate the quenching mechanism. The alternations of HSA secondary structure in the presence of CIT are demonstrated with UV-Vis, synchronous fluorescence, and CD spectra. The molecular modeling results reveal that CIT can bind with hydrophobic pocket of HSA with hydrophobic and hydrogen bond force. Moreover, an apparent distance of 3.25 nm between Trp214 and CIT is obtained via fluorescence resonance energy transfer method.

## 1. Introduction

**Figure 1:**
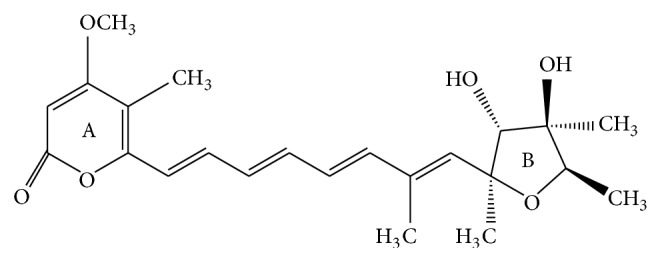
The chemical structure of CIT.

Citreoviridin (CIT, Figure 1), a toxic secondary metabolite produced by* Penicillium citreoviride* species [1], is a common contaminant of wide range of agriproducts and has a large range of toxic effects, and CIT has been associated with human health risks [2, 3]. Previous studies showed that the crude CIT extraction from* P. citreoviride* species could induce acute toxic effects in rat, guinea pig, and cat. Toxicological studies indicated that CIT was probably responsible for malignant acute cardiac beriberi [2, 4] which broke out in several rice-producing countries and regions and was characterized by many common violent symptoms, such as convulsions, tremors, respiratory arrest, ataxia, and paralysis [5–7]. Many international organizations have been working on establishing universal standards to control, regulate, and limit the CIT, and numerous countries and regions have set statutory levels for this mycotoxin.

Human serum albumin (HSA) is the most abundant protein in serum and plasma. It is capable of binding to a wide variety of endogenous and exogenous compounds, such as fatty acids, hormones, toxics, and drugs, performing as carrier and transporter [8]. HSA is the best-studied model to understand the ligand delivery process in vivo because interaction of any toxicant with HSA influences the transportation of nutrients and drugs. Owing to advantageous biochemical and toxicological properties of HSA, HSA is playing an increasing role as a toxics carrier in the toxicology setting [9]. Therefore, the knowledge of interaction mechanisms between toxics and HSA is very important for toxics research. Recently, studies have been conducted on the binding of organic contaminants or toxins to HSA, for example, arazine, ochratoxin A, methyl parathion, arsenic, perfluorooctanoic acid, patulin, deoxynivalenol, and 2-mercaptobenzimidazole [10–17].

In this work, the binding of CIT with HSA is investigated using fluorescence spectroscopy, ultraviolet-visible (UV-Vis) absorption, circular dichroism (CD) methods, and molecular modeling.

## 2. Materials and Methods

### 2.1. Materials and Solutions

HSA (fatty acid free, 96~99%) is purchased from Sigma-Aldrich and prepared in 50 mmol L^−1^ Tris-HCl (pH 7.4) at the concentration of 1.5 × 10^−5 ^mol L^−1^ and stored in the dark at 4°C. CIT (>99.0%) is provided by Fermentek (Jerusalem, Israel) and dissolved with absolute alcohol at the concentration of 1.0 × 10^−3^ mol L^−1^. Other chemicals are of analytic grade. 0.05 mol L^−1^ Tris-HCl (pH 7.4) buffer solutions are used to maintain the solution pH. 1.0 mol L^−1^ NaCl solution is used to keep the ionic strength of the buffers to 0.1 mol L^−1^. Double distilled water was used throughout.

The test solutions were prepared as follows. The 0.5 mL NaCl solution, 0.5 mL HSA solution, and appropriate amounts of CIT are added to 5.0 mL standard flasks, respectively, and then diluted to 5.0 mL with the Tris-HCl buffer solution.

### 2.2. Apparatus and Methods

#### 2.2.1. Steady-State Fluorescence Spectroscopy

Fluorescence spectra of HSA-CIT are recorded from 300 to 500 nm on F-4500 fluorescence spectrometer (Hitachi, Japan) at an excitation wavelength of 295 nm. Excitation and emission bandwidths are 5 nm. To examine the effect of temperature, spectra of HSA-CIT are collected at the temperature of 297, 303, and 308 K. Triplicate samples are measured. It should be noted that CIT shows absorbance at 295 nm and 340 nm; consequently, an inner filter effect caused some decrease in the fluorescence emission intensity. Thus, the fluorescence intensities are corrected for absorption of exciting light and reabsorption of the emitted light to decrease the inner filter effect according to [18]

(1) $$\begin{array}{c} F=F_{\text{obs}}\times 10^{\left(A_{\text{ex}}+A_{\text{em}}\right)/2}, \end{array}$$

where *F* and *F*_obs_ are corrected and observed fluorescence intensities, respectively, and *A*_ex_ and *A*_em_ are the absorption of CIT at the excitation and the emission wavelengths, respectively. The intensity of fluorescence is corrected with (1) in this study.

#### 2.2.2. Synchronous Fluorescence Spectroscopy

Synchronous fluorescence spectra of HSA in the absence and presence of increasing amount of CIT are measured with excitation and emission wavelength ranging from 200 to 500 nm at 297 K.

#### 2.2.3. UV-Vis Absorbance Spectroscopy

Absorption spectra are obtained with a UNICO UV-2802 spectrometer (Shanghai, China) at 297 K across 200–400 nm using a 1 cm quartz cell.

#### 2.2.4. CD Spectroscopy

CD spectra are measured on a JASCO J-820 automatic spectropolarimeter (JASCO, Tokyo, Japan) using a 0.1 cm cell. The buffer solution is used as a blank and automatically subtracted from the samples during scanning. Data are recorded from 200 to 250 nm with a scan speed of 100 nm·min^−1^. The concentration of HSA is maintained at 1.5 × 10^−6 ^mol L^−1^. The measurements are repeated three times.

#### 2.2.5. Molecular Modeling

The 3D structures of the ligand are constructed with standard bond lengths and bond angles using molecular modeling software SYBYL8.0 (Tripos Inc., St. Louis, USA) for Linux. Geometry optimization is implemented by using the standard Tripos force field [19, 20] with distance-dependent dielectric function and energy gradient of 0.001 kcal·mol^−1^.

Molecular docking is performed using MOE2009 for Windows (Chemical Computing Group Inc., Montreal, Canada). The available X-ray structure of HSA complexed with R-warfarin (PDB code: 1H9Z) is used in this work as the receptor. During the docking process, polar hydrogen is added to the HSA and the Kollman united atom partial charges are assigned. The grid map for site I of the HSA is centered at the middle of the IIA subdomain and is calculated with a grid spacing of 0.375 Å. Lastly, a maximum of 10 conformers were considered for CIT. In the docking calculations, all of the CIT torsional bonds are considered as free. In addition, HSA is considered as rigid and the effects of the solvent molecules are ignored. And 150 docking runs for CIT are handled. These complexes are sorted into clusters based on the root mean squares (RMS) cluster tolerance (2.0 Å used in this work) between structures. Finally, cluster 5 is identified as the preferred binding site because it has the lowest mean binding energy and the largest number of structures.

## 3. Results and Discussion

### 3.1. Analysis of Fluorescence Quenching of HSA by CIT

**Figure 2:**
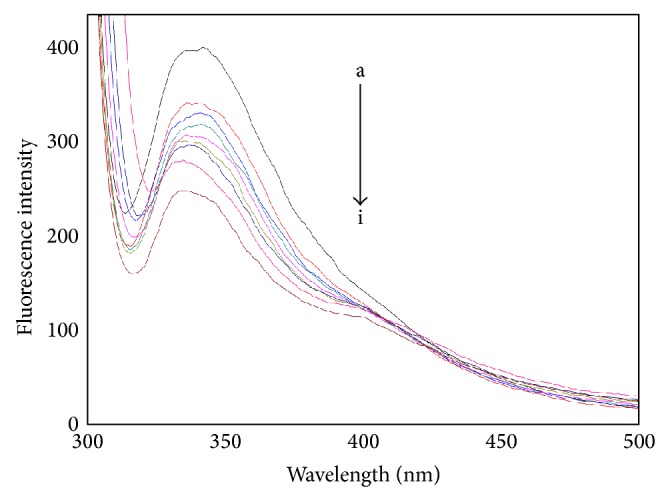
The fluorescence emission spectra of the CIT-HSA system. The concentration of HSA was 1.5 *μ*M while the CIT concentration corresponding to 0, 1.67, 3.33, 5.0, 6.67, 8.33, 10.0, and 13.3 *μ*M is from a to i, respectively. Tris buffer, pH = 7.4, *T* = 297 K, *λ*_ex_ = 295 nm.

The intrinsic fluorescence of HSA originates from tryptophan (Trp), tyrosine (Tyr), and phenylalanine (Phe) residues. Actually, the intrinsic fluorescence of HSA is mainly contributed by the Trp residue alone (Trp 214), because the Phe residue has a very low quantum yield, and the fluorescence of Tyr is almost totally quenched when it is ionized or nearby an amino group, a carboxyl of a Trp group. The fluorescence emission spectra of HSA spiked with various amounts of CIT are shown in Figure 2. When the solution is excited at 295 nm, HSA displays a strong fluorescence emission peak at 342 nm. The addition of CIT leads to a distinct decrease of the fluorescence intensity. These results indicate that CIT interacted with HSA and quenched its intrinsic fluorescence [21].

There are mainly two types of quenching mechanisms in solution: the static quenching and the dynamic quenching. They are caused, respectively, by ground-state complex formation and by diffusion; moreover, they also can be distinguished by their differing dependence on temperature and viscosity [22]. In static quenching, the complex stability and the quenching rate constants decrease with an increasing temperature. On the contrary, in dynamic quenching, higher temperatures result in faster diffusion and larger amounts of collision quenching; hence the quenching constant values rise with the temperature increasing [23]. To interpret the fluorescence quenching mechanism, the fluorescence quenching data at different temperatures is analyzed according to the Stern–Volmer equation [24]:

(2) $$\begin{array}{c} \frac{F_{0}}{F}=1+K_{\text{SV}}\left[\;Q\;\right]. \end{array}$$

In (2), *F*_0_ and *F* represent the steady-state fluorescence intensities in the absence and presence of CIT, respectively. *Q* is the CIT concentration. *K*_SV_ is the Stern–Volmer quenching constant, which is determined by linear regression of Stern–Volmer equation.  Table 1 shows the calculated *K*_SV_ and linear regression equation at different temperatures. The *K*_SV_ ascends with an increase in temperature; it can be concluded that the binding is arisen from the predominant dynamic collision.

If there are *n* substantive binding sites in the structure of HSA to accommodate the ligand molecules, the associative binding constant (*K*_*a*_) can be calculated as [24]

(3) $$\begin{array}{c} \log\left[\;\frac{F_{0}-F}{F}\;\right]=\log K_{a}+n\log\left[\;Q\;\right]. \end{array}$$

In (3), *K*_*a*_ is the binding constant and *n* is the number of binding sites. The values of *K*_*a*_ and *n* are also presented in Table 1 at different temperatures for CIT-HSA. The results illustrated that there is a strong binding force between CIT and HSA, and the number of binding sites is dependent on temperature from 297 to 308 K. The results indicate that the values of *K*_*a*_ rise with the rising temperature.

### 3.2. Thermodynamic Analysis and the Binding Force

In order to further explain the interacting forces between HSA and CIT, a thermodynamic process is considered to be responsible, which can be obtained by (4) and the Van't Hoff equation:

(4) ln⁡⁡Ka−ΔH0RT+ΔS0R,

(5) ΔG0=ΔH0−TΔS0,

where *K*_*a*_ is the binding constant at temperature *T* and *R* is gas constant. The thermodynamic parameters of CIT-HSA system determined by linear Van't Hoff equation are also listed in Table 1. From Table 1, it can be seen Δ*G* ^0^ < 0, Δ*S* ^0^ > 0, and Δ*H* ^0^ > 0. The results indicate that the binding process between CIT and HSA is spontaneous and hydrophobic interactions bonds play a major role [25].

### 3.3. Energy Transfer between HSA and CIT

**Figure 3:**
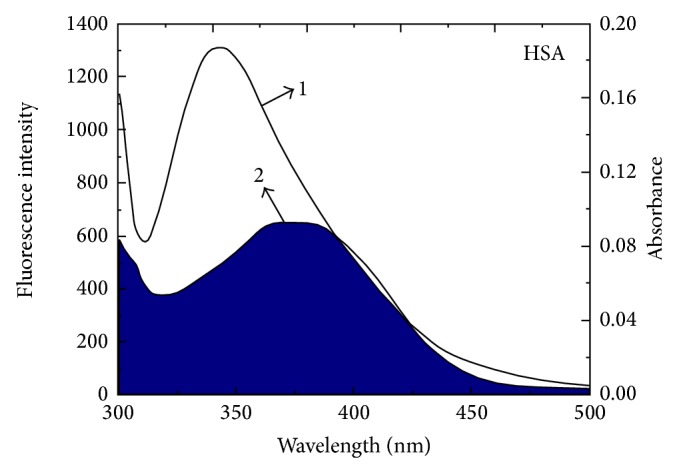
The overlap of 2 the absorption spectra of CIT and 1 the fluorescence emission spectrum of HSA. *C*_HSA_ = 1.5 *μ*M, *C*_CIT_ = 1.5 *μ*M (297 K, pH = 7.4, *λ*_ex_ = 295 nm).

Förster nonradiative energy transfer (FRET) technique has been successfully used to probe biological systems to study the structure, conformation, spatial distribution, and assembly of complex proteins [26]. The efficiency (*E*) of energy transfer between the donor and the acceptor can be calculated by the following equations:

(6) E1−FF0=R06R06+r6,R068.8×10−25K2n−4ϕJ,J∑Fλελλ4Δλ∑FλΔλ,

where *F*_0_ and *F* are the fluorescence intensities of HSA before and after addition of CIT, respectively. *R*_0_ is the critical distance when the efficiency of excitation energy transferred to the acceptor is 50% and *r* is the binding distance between donor and receptor; *K* ^2^ is the spatial orientation factor of the dipole; *n* is the refractive index of the medium; *ϕ* is the fluorescence quantum yield of the donor; *J* is the overlap integral of the fluorescence emission spectrum of the donor with the absorption spectrum of the acceptor; *F*(*λ*) is the corrected fluorescence intensity of the donor in the wavelength range from *λ* to *λ* + Δ*λ*; and *ε*(*λ*) is the extinction coefficient of the acceptor at *λ*. From the overlapping of the emission spectra of HSA and the absorption spectra of CIT (Figure 3), the calculated *J* value is 6.68 × 10^−15 ^cm^3^·L·mol^−1^ for HSA. In present case, *K* ^2^ = 2/3, *n* = 1.336, and *ϕ* = 0.118 [27]. Thus, from (6), *R*_0_ is equal to 3.36 nm and *r* is equal to 3.25 nm. Obviously, the Trp214 to CIT distance is less than 8 nm and within 0.5*R*_0_ < *r* < 1.5*R*_0_ [28]. These results suggest that the energy transfer from HSA to CIT can occur with high probability [29].

### 3.4. Change of HSA Secondary Structure Induced by CIT

#### 3.4.1. UV-Vis Spectra

**Figure 4:**
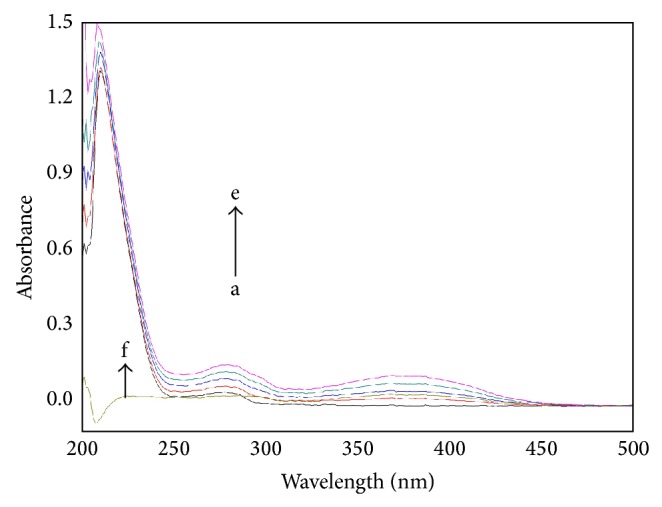
UV absorption spectra of the CIT-HSA. The concentration of HSA was 1.5 *μ*M while the CIT concentration corresponding to 0, 3.33, 6.67, 10, and 13.3 *μ*M is from a to e, respectively. *f* [HSA] = 0 *μ*M, [CIT] = 15.0 *μ*M. Tris buffer, pH = 7.4, *T* = 297 K.

Figure 4 shows the UV-Vis absorption spectra of HSA in the absence and presence of CIT. Three absorption peaks at 210, 277, and 367 nm are observed in Figure 4. The intensities of these peaks rise upon the addition of CIT. The absorption of HSA at 210 nm represents the content of *α*-helix in the protein [30]. The peak at 210 and 280 nm will be found to slightly shift towards shorter wavelengths with the increasing amounts of CIT, which indicate a decrease of *α*-helix and tryptophan (Trp) residue is exposed to a more hydrophobic environment.

#### 3.4.2. Synchronous Fluorescence Spectra

**Figure 5:**
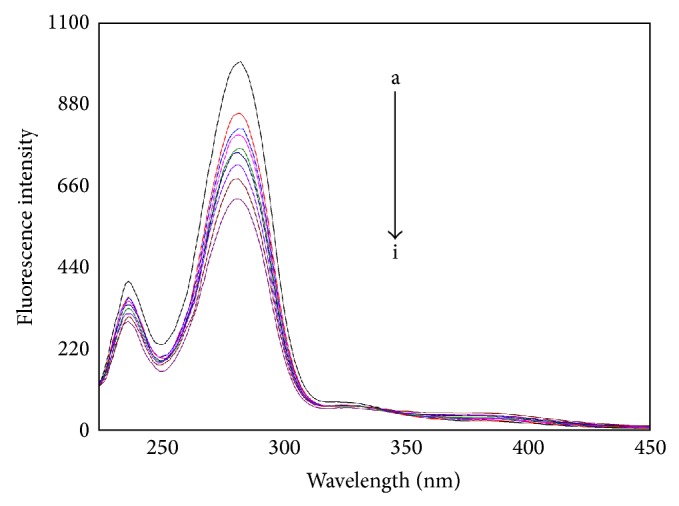
The synchronous fluorescence spectra of CIT-HSA. The concentration of HSA was 1.5 *μ*M while the CIT concentration corresponding to 0, 1.67, 3.33, 5.0, 6.67, 8.33, 10.0, and 13.3 *μ*M is from a to i, respectively. Tris buffer, pH = 7.4, *T* = 297 K, Δ*λ* = 60 nm.

According to Miller [31], when Δ*λ* is set at 60 nm, the shift of the maximum emission wavelength reveals the alteration of polarity microenvironment around Trp residues. The synchronous fluorescence spectra of HSA in the absence and the presence of CIT are shown in Figure 5. The fluorescence intensity of Trp residue reduces and the peak position shows a slight blue shift. The results are in agreement with the previous fluorescence spectra. The results suggest that the polarity microenvironment around Trp residue decreases and Trp residue exposes to a more hydrophobic environment.

#### 3.4.3. CD Spectra

CD spectroscopy is a powerful method in structural biology that has been used to examine proteins, polypeptides, and peptide structures since the 1960s. Because the spectra of these molecules in the far ultraviolet (UV) regions are dominated by the n-*π* ^∗^ and *π*-*π* ^∗^ transitions of amide groups and are influenced by the geometries of the polypeptide backbones, their spectra are reflective of the different types of secondary structures present.

**Figure 6:**
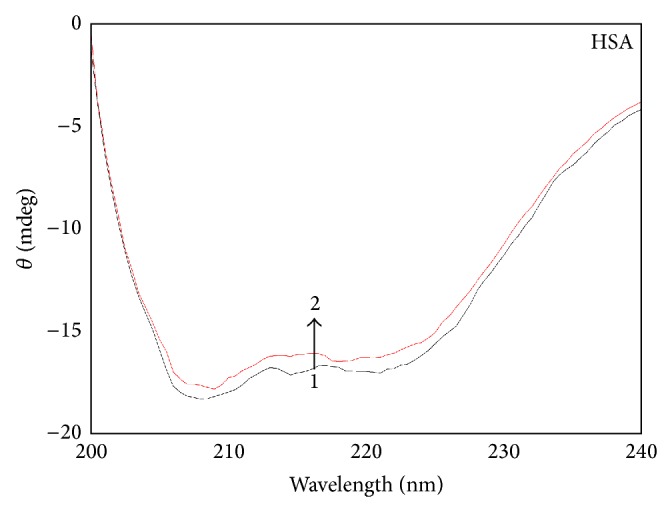
The CD spectra of the CIT-HSA system. CIT concentration was 0 *μ*M 1 and 3.0 *μ*M 2, respectively. *C*_HSA_ = 1.5 *μ*M, pH  =  7.4, *T* = 297 K.

To confirm the influence of CIT binding on the secondary structure of HSA, CD spectra are performed in the presence of different concentrations of CIT. As shown in Figure 6, the CD spectra of HSA have two negative bands at 208 and 220 nm, which are typical characterization of *α*-helix structure in HSA [32]. According to the report, the negative peaks between 208 and 209 nm and 222 and 223 nm are contributed to n-*π* ^∗^ transfer for the peptide bond of *α*-helix [33]. From Figure 6, the two negative bands intensities of HSA at 208 and 220 nm increase with negative Cotton effect by the adding of CIT, which indicate the considerable changes in the protein secondary structure. The similarity between the CD spectra shapes of free HSA and CIT-HSA suggests that structure of HSA in the CIT-HSA complex is also predominantly *α*-helix. The *α*-helix contents of free and combined HSA are calculated from mean residue ellipticity values using SELCON3 program that is provided by the DichroWeb website (http://dichroweb.cryst.bbk.ac.uk/html) [34–36].

The results indicate that the *β*-sheet structure containing the HSA rises from 7.90 to 8.52%, the *β*-turn structure content rises from 18.6 to 19.1%, the random coil structure content rises from 21.9 to 22.7%, and the *α*-helix structure content lowers from 51.6 to 49.7% when the molar comcentration ratio of CIT to HSA increases from 0 : 1 to 2 : 1, respectively. The decrease of *α*-helical structures indicates that the binding of CIT with HSA causes its conformational changes and *α*-helical stability loss.

### 3.5. Molecular Modeling

**Figure 7:**
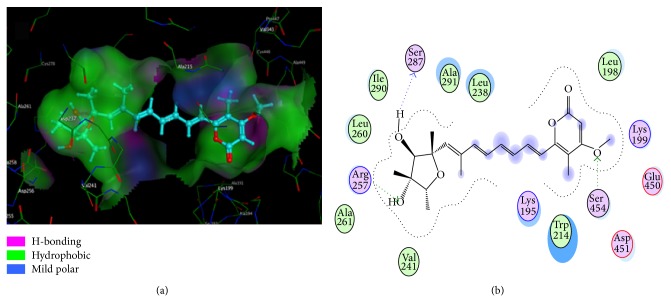
(a) The interaction mode between CIT and HSA. (b) The projection of 7(a). The HSA residues are represented by lines and the CIT structure is represented by a ball and stick model. The hydrogen bond between the CIT and HSA is represented by a dashed line.

The crystal structure analyses revealed that HSA contains the following three domains that are structurally similar (I–III): I (residues 1–195), II (196–383), and III (384–585). The principal ligand binding site regions on HSA are located in the hydrophobic cavities subdomains IIA and IIIA, which are consistent with sites I and II (according to the terminology of Sudlow et al.) [36]. It is important to note that Trp214 is in subdomain IIA. Docking results are listed in Figures 7(a) and 7(b). As shown in Figure 7, the CIT molecule is surrounded by the hydrophobic residues, such as Ala291, Leu238, Leu198, Trp214, Val241, Ala261, Leu260, and Ile290, and the two rings are not coplanar. Therefore, it suggests that hydrophobic force is the main interaction force in the binding of CIT to HSA, which is supported by the thermodynamic analysis. In addition, the two -OHs of the furan ring and a -OCH_3_ of the pyran ring form three hydrogen bonds with Ser287 (bond length 1.75 Å), Arg257 (bond length 1.73 Å), and Ser454 (bond length 2.62 Å), respectively. The docking results indicate that hydrophobic interactions and hydrogen bonding interactions exist between CIT and HSA. Furthermore, several polar residues are found near CIT molecule, which play a subordinate role in stabilizing CIT molecule through electrostatic interactions.

## 4. Conclusions

In the present work, the interaction of CIT with HSA is investigated by optical spectroscopy technique (steady-state fluorescence, UV-Vis absorption, and CD) and molecular modeling. Experimental results suggest that CIT can bind with HSA and quench the fluorescence of HSA. In addition to thermodynamic parameters, the values of binding constant and the number of binding sites of the CIT-HSA system are determined. It is found that the hydrophobic interactions and hydrogen bond forces play a major role in the binding of CIT with HSA. The average binding distance between donor and acceptor molecules is found to be 3.25 nm for HSA-CIT systems from FRET theory. Furthermore, docking calculations find CIT to be located in the hydrophobic pocket of HSA within subdomain IIA. The results provide significant information for the understanding of CIT transportation process in vivo and are expected to provide some useful information for researching toxicology of CIT.

## Figures and Tables

**Table 1:** The Stern–Volmer equations, *K*_SV_, *K*_*a*_, *n*, and relative thermodynamic parameters of the system of CIT-HSA.

*T* (K)	The Stern–Volmer			The modified Scatchard			Δ*G* (KJmol^−1^)	Δ*H* (KJmol^−1^)	Δ*S* (Jmol^−1^K^−1^)
	Liner equation	*R* ^2^	*K*_SV_ (10^4^ L mol^−1^)	*R* ^2^	*K*_*a*_ (10^3^ L mol^−1^)	*n*			
297	*Y* = 1.1279 + 2.650 × 10^4^ [*Q*]	0.9960	2.650	0.9851	0.09541	0.4793	−25.13	19.64	150.8
303	*Y* = 1.0610 + 3.040 × 10^4^ [*Q*]	0.9880	3.040	0.9730	0.4937	0.6286	−26.04		
308	*Y* = 0.9254 + 3.526 × 10^4^ [*Q*]	0.9927	3.526	0.9977	208.9	1.3792	−26.79		

## Abbreviations

CIT: Citreoviridin
HSA: Human serum albumin
CD: Circular dichroism
Trp: Tryptophan
Tyr: Tyrosine
Phe: Phenylalanine.
